# Modulation of defense genes and phenolic compounds in wild blueberry in response to *Botrytis cinerea* under field conditions

**DOI:** 10.1186/s12870-023-04090-5

**Published:** 2023-02-28

**Authors:** Joel Abbey, Sherin Jose, David Percival, Laura Jaakola, Samuel K. Asiedu

**Affiliations:** 1 Department of Plant, Food, and Environmental Sciences, Faculty of Agriculture, Dalhousie University, 50 Pictou Road, P.O. Box 550, Truro, NS B2N 2R8 Canada; 2 Department of Arctic and Marine Biology, The Arctic University of Norway, Tromso, Norway; 3 NIBIO, Norwegian Institute of Bioeconomy Research, P.O. Box 115, NO‑1431, Ås, Norway

**Keywords:** Blueberry, *Botrytis cinerea*, Real-time RT-PCR, Gene expression, Phenolics, HPLC–DAD

## Abstract

Botrytis blight is an important disease of wild blueberry [(*Vaccinium angustifolium (Va)* and *V. myrtilloides (Vm)*)] with variable symptoms in the field due to differences in susceptibility among blueberry phenotypes. Representative blueberry plants of varying phenotypes were inoculated with spores of *B. cinerea.* The relative expression of pathogenesis-related genes (*PR3*, *PR4*), flavonoid biosynthesis genes*,* and estimation of the concentration of ten phenolic compounds between uninoculated and inoculated samples at different time points were analyzed. Representative plants of six phenotypes (brown stem *Va,* green stem *Va*, *Va* f. nigrum, tall, medium, and short stems of *Vm*) were collected and studied using qRT-PCR. The expression of targeted genes indicated a response of inoculated plants to *B. cinerea* at either 12, 24, 48 or 96 h post inoculation (hpi). The maximum expression of *PR3* occurred at 24 hpi in all the phenotypes except *Va* f. nigrum and tall stem *Vm*. Maximum expression of both *PR* genes occurred at 12 hpi in *Va* f. nigrum. Chalcone synthase*,* flavonol synthase and anthocyanin synthase were suppressed at 12 hpi followed by an upregulation at 24 hpi. The expression of flavonoid pathway genes was phenotype-specific with their regulation patterns showing temporal differences among the phenotypes. Phenolic compound accumulation was temporally regulated at different post-inoculation time points. M-coumaric acid and kaempferol-3-glucoside are the compounds that were increased with *B. cinerea* inoculation. Results from this study suggest that the expression of *PR* and flavonoid genes, and the accumulation of phenolic compounds associated with *B. cinerea* infection could be phenotype specific. This study may provide a starting point for understanding and determining the mechanisms governing the wild blueberry-*B. cinerea* pathosystem.

**Supplementary Information:**

The online version contains supplementary material available at 10.1186/s12870-023-04090-5.

## Introduction

Wild blueberry [*Vaccinium angustifolium* (Aiton) Rydb. *(Va)* and *V. myrtilloides* (Michx.) House (*Vm*)] is an important crop and a leading horticultural commodity in Eastern Canada and Maine, USA. Wild blueberries are native to North America and commercial fields are developed from forested areas or abandoned farmlands. Due to their wild nature and inherent presence in forest areas, fields are made up of different species with differences in ploidy level and varying phenotypes within and between species. Commercial fields mostly consist of tetraploid *Va* (~ 70–80% on a surface area basis), diploid *Vm* (~ 10–20%), and some other *Vaccinium* spp. hybrids [1]. *Vm* is a densely velvety with heights ranging from 10 – 60 cm. The leaf margins are complete and have bright blue fruit. *Va*, on the other hand, is verrucose with heights ranging from 5–40 cm. Their leaf margins are serrated and produce bright, blue-colored fruit [2]. *Va* f. nigrum is a subspecies of *Va*, with bright pink flowers and dark/blackish fruits.

Several diseases affect wild blueberries, including Septoria leaf spot (*Septoria* spp.), Botrytis blight (*Botrytis cinerea* Pers.:Fr) and Monilinia blight (*Monilinia vaccinii-corymbosi* (Reade) Honey) [3, 4]. Among these diseases, Botrytis blight has been a major problem with far-reaching economic implications. *Botrytis cinerea* infects the blueberry plant’s aerial parts, particularly the flowers or entire inflorescences [5]. Infected flowers exhibit a brown, water-soaked appearance that extends to cover the whole flower. Dead flowers are usually covered with the characteristic dense greyish mycelia and spores of *B. cinerea*. Infections can spread quickly through the flowers and often destroy the entire inflorescence. The susceptibility of flowers to the fungus is dependent on the developmental stage of the flower. The flower is most susceptible at the F7 floral stage when the corolla is fully opened [5, 6]. Botrytis blight can be a severe disease, however, the effect on fields varies extensively due to differences in susceptibility among the various phenotypes. Over the years, minimal damage from Botrytis and Monilinia blights in *Vm* has been reported [6–8]. *Vm* has been identified as a potential source of blight resistance in breeding programs due to its tolerance as stated in the study by Ehlenfeldt and Stretch [7]. In a recent study, Abbey et al. [6] indicated that *Va* was the most susceptible to *B. cinerea* followed by *Va* f. nigrum whereas *Vm* was found to be least susceptible.

Presently, Botrytis blight management is primarily dependent on chemical fungicide application. However, growing concerns about environmental safety, the development of fungicide resistance among the pathogen population, and rising production costs make it difficult to rely on this strategy indefinitely. Given this, alternative disease management that reduces the challenges posed by chemical fungicides is critical. Integrating plants’ natural defense mechanisms into disease management programs could be a viable and long-term disease management strategy. Therefore, understanding the molecular basis of wild blueberry response to pathogenic and non-pathogenic microbes through gene expression analysis could contribute to understanding the disease resistance mechanism in wild blueberry.

Plants are known to accumulate proteins and biochemical compounds in response to biotic and abiotic stresses to delay or reduce the impact of these stresses on them [9, 10]. Generally, pathogenesis-related (PR) proteins are induced upon infection and are associated with host defense machinery to limit pathogen progress [11]. Among the biochemical compounds, flavonoids are known to play an important role in plant defense against various stresses [12]. Many studies have been conducted on the host response of various plants to various pathogens including *Botrytis* spp*.* Cui et al. [13] reported a high accumulation of transcripts of the genes encoding for various *PR* proteins in leaves of *Lilium regale* infected with *Botrytis elliptica* (Berk.) Cooke. Depending on the type of pathogen involved PR genes expressed will vary. For instance, the expression of *PR* 1, 2, and 5 are mostly associated with biotrophic and hemibiotrophic pathogens [14] whereas *PR* 3, 4, and 12 are associated with necrotrophic pathogens such as *B. cinerea* [15, 16].

Similar to some *PR* proteins, several genes involved in the phenylpropanoid pathway, their related compounds that possess antimicrobial capabilities are accumulated during pathogen infection [17, 18]. For instance, an increase in the expression of flavonoid genes (CHS, chalcone synthase and ANS, anthocyanidin synthase), and related phytoalexin compounds (catechin and quercetin) in *B. cinerea* and endophyte *Paraphaeosphaeria* sp. inoculated bilberry leaves have been reported [19]. Also, an interaction between grapevine flower and *B. cinerea* resulted in a rapid defense reaction involving the activation of genes associated with the accumulation of antimicrobial proteins, polyphenols, and cell wall reinforcement [20]. Additionally, non-pathogenic, or beneficial microbes have been reported to alter the expression of these defense responses in plants [21, 22]. There are many studies on plant disease response from different host–pathogen interactions, however, there is no such study on the molecular and biochemical changes induced in wild blueberry during their interaction with *B. cinerea*.

In this study, we investigated the wild blueberry defense responses against *B. cinerea* through the expression levels of selected *PR* and flavonoid biosynthesis pathway genes known to be involved in plant defense responses. We also investigated some biochemical changes that occur during an interaction between wild blueberry and *B. cinerea*.

## Materials and method

### Experimental design

**Fig. 1:**
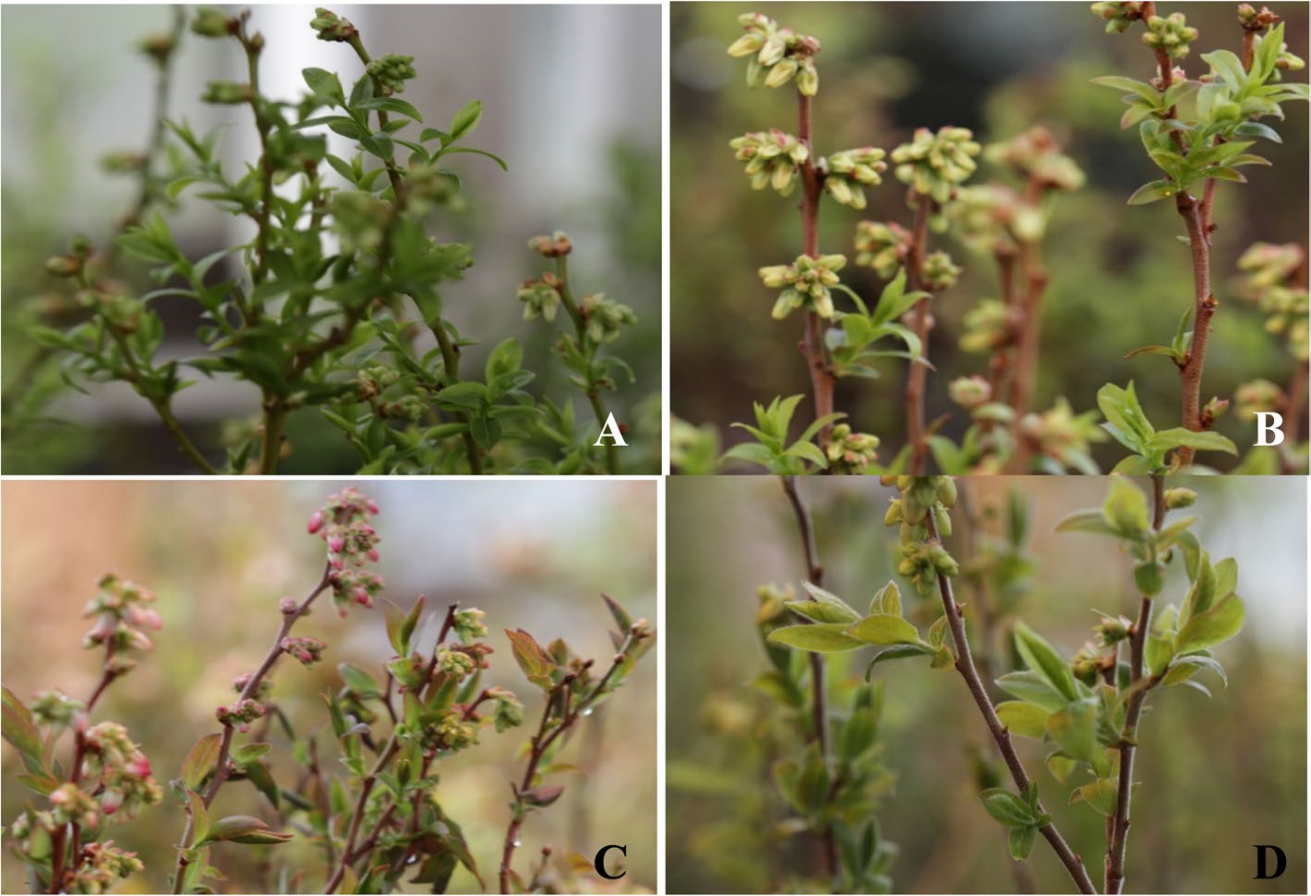
*V. angustifolium* green stem (**A**), *V. angustifolium* brown stem (**B**), *V. angustifolium* f. nigrum (**D**) and *V. myrtilloides* (**C**)

Representative plants of six phenotypes which consisted of 3 *Vaccinium angustifolium* (*Va* brown stem, *Va* green stem, *Va* f. nigrum) and 3 *Vaccinium myrtilloides* (*Vm* short, *Vm* medium, and *Vm* tall stem) were selected from a commercial wild blueberry field, NS, Canada in June 2019 (Fig. 1). The commercial field used belonged to the Bragg Lumber company who was part of the collaborative research under which this study was conducted. *Vm* plant height was classified as short (< 15 cm), medium (15 – 25 cm), and tall (> 25 cm). In the fields, short stem *Vm* has been observed to be more tolerant to Botrytis blight and Monilinia blight, hence the inclusion of different heights of *Vm*. The response of these phenotypes to *B. cinerea* inoculation at the F7 stage of floral growth (corolla fully opened) was assessed. Three biological replicates (each patch size was 1 m × 2 m area) were selected for each phenotype and each replicate was separated into two, 0.5 × 1 m sample areas. One day before inoculation, one sample area within each replicate was sprayed with the fungicide, Switch® (cyprodinil and fludioxonil, 625 g a. i./L) to serve as the check/control for generating a ΔCt calibrator for the ΔΔCt gene expression analysis [23].

### Inoculation and sample collection

**Fig. 2:**
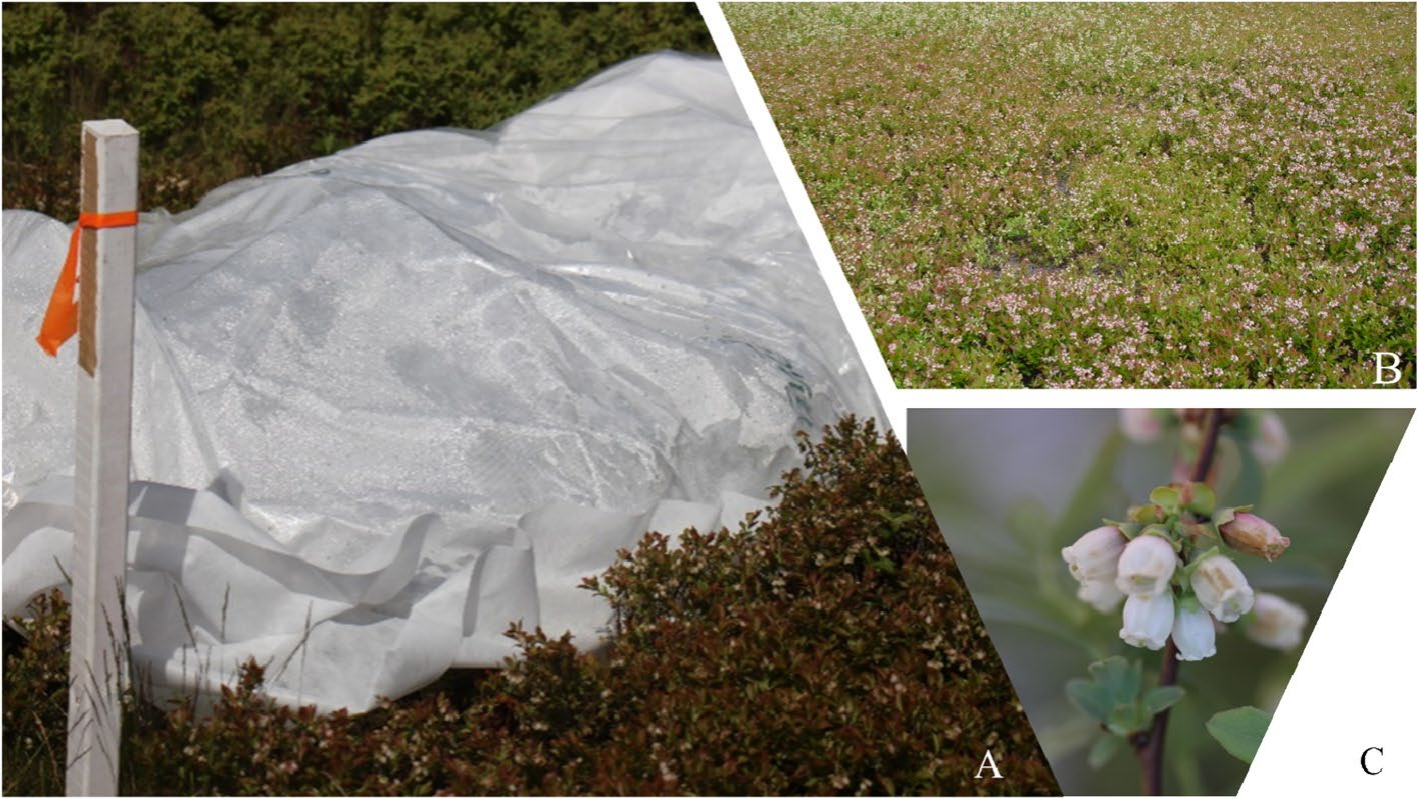
Experimental setup on a commercial wild blueberry field. **A** Inoculated patch in with a row cover with a 2 mm plastic film to create a humid condition for infection to occur, **B** A patch of wild blueberry in their natural growing habit on a commercial field, and **C** Infected wild blueberry flower at F7 flower stage (Corolla fully opened)

Distilled water-based spore suspension (10^6^ conidia mL^−1^) was prepared from a two-week-old single spore *B. cinerea* culture isolated grown on potato dextrose agar (PDA). The *B. cinerea* was isolated from infected *Va* floral tissue and identified based on its morphological characteristics under the microscope [24]. The spore concentration was estimated using a hemocytometer (BLAUBRAND® Neubauer) and adjusted to 1 × 10^6^ conidia mL^−1^ and Tween 20 (0.04%) was added to the suspension prior to inoculation. The 10^6^ conidia mL^−1^ concentration was tested before the experiment to ensure that the concentration was sufficient to adequately cause infection. The spore suspension was applied to the plants in the remaining sample areas of each plot that did not receive the fungicide within the replicate using a hand-held pump sprayer to produce very fine evenly distributed droplets on each plant to the point of runoff. The plants were immediately covered with a 2 mm plastic film and row cover (DeWitt Plant & Seed Guard, Halifax seed, NS) to provide favorable conditions (100% RH) for 48 h (Fig. 2). Prior to inoculation, floral tissues (whole flowers) were harvested to represent 0 h before inoculation or basal expression (0 hbi). Post inoculation, flower tissues were harvested at 12-, 24-, 48-, and 96-h (hpi). For every sample collection, flowers from 20 plants within each replicate were harvested and pooled together for RNA extraction. The samples were immediately flash frozen in liquid nitrogen and later preserved in -80 °C for gene expression and chemical analyses.

### RNA Extraction and cDNA synthesis

Total RNA was isolated from the floral tissue using Qiagen RNeasy Plant kits following the manufacturer’s instruction (QIAGEN, Valencia, CA, USA). Genomic DNA contamination was removed by on-column DNase I digestion (Qiagen Inc., Valencia, CA, USA). The concentration and RNA purity was assessed based on an absorbance ratio of 1.8 to 2.0 at 260/280 nm and ≥ 2.0 at 260/230 using the Biotek Synergy H1 Hybrid Multi-Mode Reader (BioTek Instruments Inc., Winooski, VT, USA). DNA-free total RNA (1 µg) was used for the cDNA synthesis using MultiScribe™ Reverse Transcriptase from the High-Capacity cDNA Reverse Transcription Kit (Applied Biosystems, CA, USA) in a 20 µL reaction following the manufacturer’s instruction. The MultiScribe™ reaction mix includes random primers to make cDNAs. The final cDNA products were diluted 20-fold before use in real-time PCR.

### Quantitative real-time PCR (qRT-PCR) analysis

Quantitative RT-PCR (qPCR) analysis of cDNA was carried out in a 96-well rotor in BIO-RAD CFX Connect Real-Time System using BioRAD SsoAdvanced Universal SYBR Green Supermix (BioRad Laboratories Inc., CA, USA) in a 10 µL reaction. Each 10 µL reaction comprised 5 µL SYBR Green supermix, 1 µL H_2_O, 2 µL cDNA, and 1 µL forward and reverse primers (10 nM) for each gene of interest. The qPCR parameters used are as follows: 95 °C for 3 min, 35 cycles each at 95 °C for 10 s, and 60 °C for 20 s. Each qPCR reaction was carried out in three technical replicates and a no-template controls (NTC) with glyceraldehyde-3-phosphate dehydrogenase (GAPDH) as a reference gene [25]. Gene sequences were retrieved from *V. corymbosum* database (www.vaccinium.org) and the National Center for Biotechnology Information (NCBI; www.ncbi.nlm.nih.gov) to design primers for this study. Specific primers were designed with Primer Premier 5.0 (Premier Biosoft International, California, USA) and analyzed with different bioinformatics tools (BioEdit/ Clustal w/BLAST/ Primer Premier 5.0) (Supplementary file, Table S1). Relative quantification of genes was obtained using the ΔΔCt method. In brief, the Ct values of target genes were normalized to the reference gene (GAPDH) (ΔCT = Ct _target_—Ct _GAPDH_) and compared with a calibrator (ΔCT = Ct _sample_—Ct _control_). Relative expression (RQ) of the genes was calculated by the formula 2^− ΔΔ CT^ method using Ct value [23].

### HPLC–DAD analysis of flavonoids and hydroxycinnamic acids

#### Chemicals and standards preparation

External standards of caffeic acid, neochlorogenic acid, catechin, procyanidin B2, quercetin-3-galactoside, m-coumaric acid, p-coumaric acid, and quercitrin (quercetin 3-rhamnoside) were purchased from Sigma- Aldrich, Inc. (St. Louis, MO, USA). Chlorogenic acid was purchased from MP medicals, France, and kaempferol-3-glucoside was obtained from the HWI group (Rheinzaberner, Germany). Analytical grade methanol, sodium fluoride (NaF), and formic acid (> 95%) were purchased from Merck® (Bengaluru, India). HPLC-grade water was obtained from a Milli-Q System with a resistivity of 18.2 mΩ (Millipore, Billerica, MA, USA).

Calibration standards were prepared by an appropriate dilution of stock solutions with 50% methanol. Nine different concentrations of each compound within 0.01—200 µg/mL for all the compounds were prepared to generate calibration curves. Standard curves were generated using linear regression (R^2^ of each standard curve was > 0.99).

#### Extraction and analysis of phenolic compounds

Phenolic compounds were extracted and subsequently analyzed by reverse-phase high performance liquid chromatography—diode-array detection (HPLC–DAD) as described by Tomás-Barberán et al. [26] and Villarino et al. [27] with modifications. Frozen samples collected at 48- and 96-h post-inoculation were ground to a fine powder in liquid nitrogen for extraction. Ground material (0.2 g) was extracted with 5.0 mL extraction solution (2% Formic acid 80% methanol containing 2 mM NaF to inactivate polyphenol oxidases and prevent phenolic degradation) for 60 min at 8 ºC in the dark. The extract was centrifuged at 4,300 rpm for 15 min at 4 °C and the supernatant was transferred into a clean tube. The extraction was repeated a second time on the residue from the first extraction after which the two supernatants were combined and 1 mL aliquot was filtered through a 0.45 μm nylon filter for analysis.

Phenolic compound compositions were determined from the filtrate using Waters® e2695 HPLC with auto injector equipped with a 2998 photodiode array detector (Waters Corp., Milford, U.S.A.) equipped with a degasser. A Phenomenex Kinetex™ C_18_ column [250 X 4.6 mm (inner diameter); particle size, 5 μm] was used to separate the phenolic compounds at a temperature of 25 °C. The mobile phases were water (A), and methanol (B) both of which contained 0.5% formic acid to increase peak resolution. The gradient used for eluent A was 100% (0–5 min), 85% (5–20 min), 50% (20–25 min), 30% (25–30 min), 0% (30–40 min), and 100% (40–60 min). The determination was conducted at a flow rate of 1.0 mL/min. Phenolic compounds were identified and quantified by comparing their retention times with those of their respective external standards at wavelengths of 280, 302 and 355 nm (Supplementary file, Table S2).

### Statistical analysis

Gene expression and phenolic compound data were analyzed using a two-way ANOVA with phenotype and time as fixed factors and replicate as the random factor. The PROC GLIMMIX procedure of SAS (version 9.4, SAS Institute, Inc., Cary, NC) was used for the analysis. The least significant difference (LSD) test was used for multiple means separation at α = 0.05.

## Results

### Pathogenesis-related genes

**Fig. 3:**
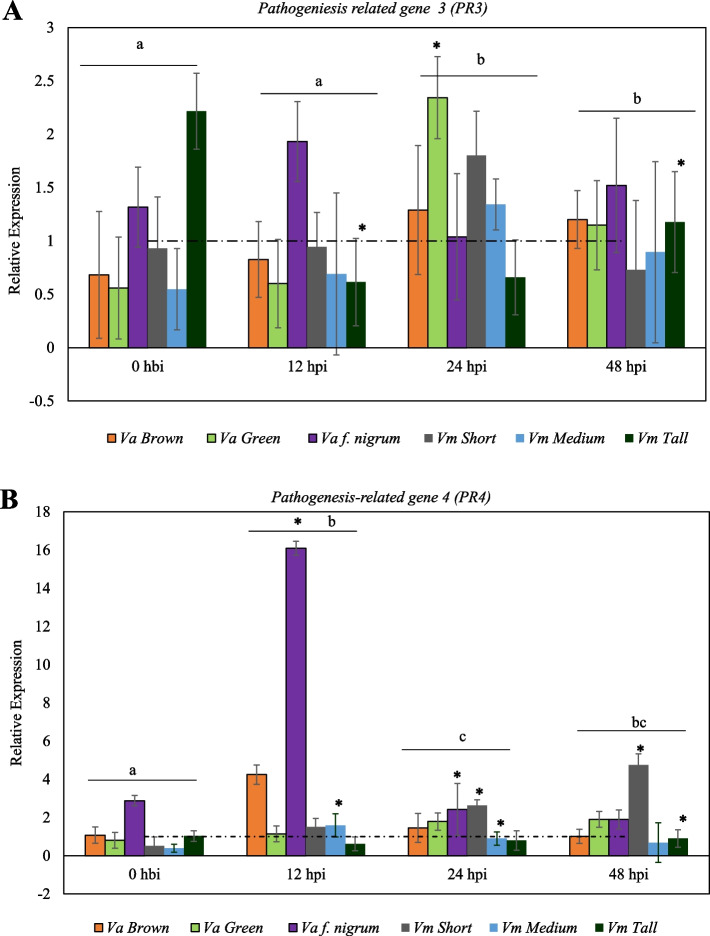
Expression pattern of pathogenesis-related genes in wild blueberry phenotypes (*V. angustifolium* and *V. myrtilloides*) in response to *Botrytis cinerea* infection. **A** Relative expression of *PR3*. **B** Relative expression of *PR4.* Expression of each gene is shown as a fold change in infected samples relative to their respective uninfected check/control from the same time point. Results are reported as means ± standard error of three biological replicates. Asterisks (*) indicate **s**ignificant difference between infected plants and their basal expression (0 h before inoculation, hbi). Post inoculation time points (hbi/hpi) with the same letters on the horizontal bar are not significantly different from each other at α = 0.05. Broken horizontal line at onefold relative expression represents the calibrator

The expression of pathogenesis-related genes was observed at the early (12 hpi) phase of the infection process in all the phenotypes except *Vm* tall stem. However, the maximum expression levels of these PR genes varied among the *Va* phenotypes*.* The maximum expression of *PR* genes was early in *Va* f. nigrum but delayed in green and brown stem *Va* (Fig. 3a, b). The expression of *PR3* and *PR4* in both brown and green stems of *Va* was observed at 12 hpi, however, maximum *PR3* expression was observed at 24 hpi while maximum *PR4* expression was observed at 12 hpi in brown stem *Va* (Fig. 3a, b). Similarly, in the green stem *Va,* significant upregulation of *PR3* was observed at 24 hpi (Fig. 3b). In *Va* f. nigrum, both *PR3* and *PR4* were highly expressed, however, maximum *PR4* expression was observed at 12hpi (Fig. 3b). In the *Vm* phenotypes, the levels of expression varied between the short and medium stems phenotypes. In the short stem *Vm,* a noticeable expression of *PR3* was observed at 24 hpi. In the medium stem *Vm, PR3* expression was maximum at 24 hpi whereas, *PR4* was expressed at 12 hpi. There was no remarkable expression of these *PR* genes in the tall stem *Vm* but rather a decrease in their expression after inoculation (Fig. 3a, b).

At the phenotype level, the expression of pathogenesis related genes revealed high expression of *PR3* (*p* = 0.0119) and PR4 (*p* = 0.0001) in *Va* f. nigrum while tall stem *Vm* had the least expression. Regarding temporal expression, the expression of both *PR* genes was higher at 24 and 48 hpi (Fig. 1a, b).

### Flavonoid pathway genes

**Fig. 4:**
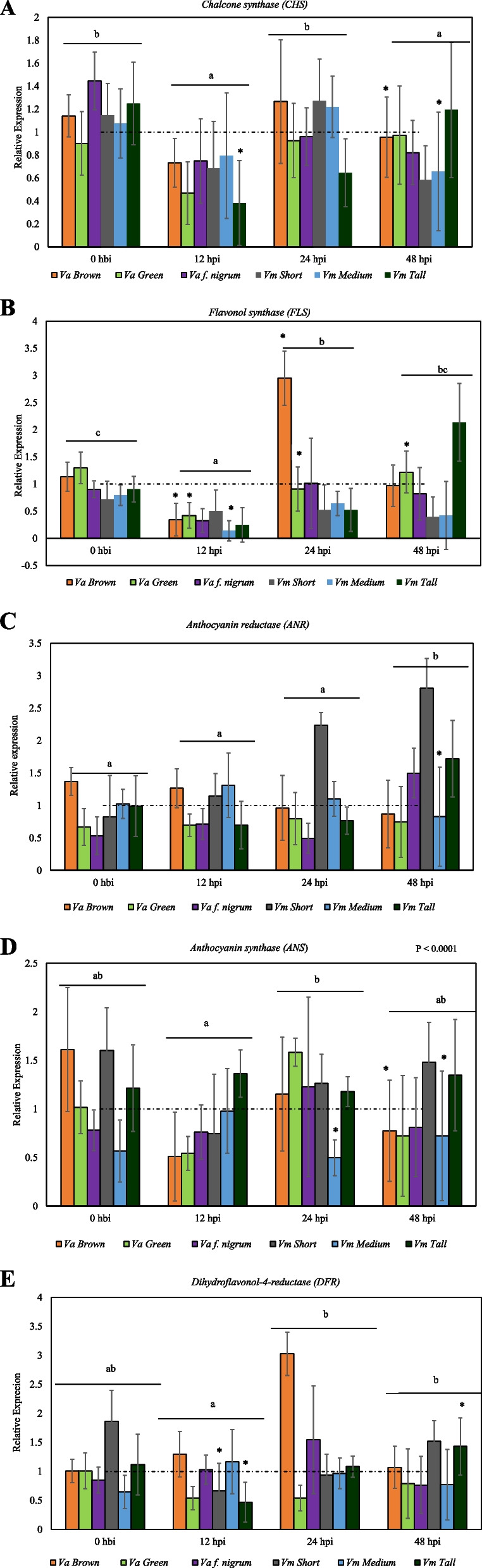
Expression pattern of flavonoids biosynthesis genes in wild blueberry phenotypes (*V. angustifolium* and *V. myrtilloides*) in response to *Botrytis cinerea* infection. **A** Chalcone synthase (*CHS*);** B** Flavonol synthase(*FLS);***C** Anthocyanin reductase (*ANR*); **D** Anthocyanin synthase (*ANS*); **E** Dihydroflavonol-4-reductase (*DFR*). Expression of each gene is shown as a fold change in infected samples relative to their respective uninfected check/control from the same time point. Results are reported as means ± standard error of three biological replicates. Asterisks (*) indicate** s**ignificant difference between infected plants and their basal expression (0 h before inoculation, hbi). Post inoculation time points (hbi/hpi) with the same letters on the horizontal bar are not significantly different from each other at α = 0.05. Broken horizontal line at onefold relative expression represents the calibrator

The expression of the flavonoid pathway genes chalcone synthase (*CHS),* flavonol synthase *(FLS)* and anthocyanin synthase *(ANS)* decreased in the early stages (12 hpi) of infection in all three *Va* phenotypes followed by a rise in expression. Although there was an increase in expression levels of *CHS* at 24 hpi, it was not significantly different from the basal expression (0 hbi) in the brown and green stem *Va* (Fig. 4a). The expression of *FLS* was higher in the brown stem at 24 hpi whereas it was not significantly different from the basal expression in *Va* f. nigrum. The expression of *FLS* in the green stem *Va* was similar to the basal expression at 48 hpi (Fig. 4b). *ANS* expression was maximum at 24 hpi in the green stem *Va* and *Va* f. nigrum (Fig. 4d). An increased expression of anthocyanin reductase (*ANR)* in *Va* f. nigrum up to 48 hpi was observed (Fig. 4c). Dihydroflavonol-4-reductase* (DFR)* expression was early (12 hpi) in brown stem *Va* and *Va* f. nigrum with the maximum expressions at 24 hpi (Fig. 4e). In the three *Vm* phenotypes, there was a decrease in *CHS* expression at 12 hpi (Fig. 4a). A decrease in the expression of *FLS* in short and medium stem *Vm* was observed. A decrease in *FLS* expression in tall stem *Vm* at 12 hpi followed a steady rise in expression up to 48 hpi was observed (Fig. 4b). *ANR* exhibited an increased expression in all three *Vm* phenotypes. There was an early response (12 hpi) of *ANR* in short and medium stem *Vm*. However, the *ANR* expression in the medium and short stem *Vm* peaked at 12 and 48 hpi respectively. An increase in *ANR* which peaked at 48 hpi was observed in the tall stem *Vm* (Fig. 4d). *ANS* and *DFR* decreased at 12 hpi in short stem *Vm*, nonetheless, there was an increase of both genes at 24 and 48 hpi (Fig. 4d, e). On the contrary, there was an increase in *ANS* and *DFR* expression in the medium stem *Vm* at 12 hpi. *ANS* showed similar expression pattern in both medium and tall stem *Vm*. However, the expression at 12 hpi was not significantly different from the basal expression (Fig. 4d, e).

At the phenotype level, no significant difference was observed with *ANR, ANS* and *DFR*. However, *Va* f. nigrum had a significantly high expression of *CHS* (*p* = 0.0041) whiles brown stem *Va* had a significantly high expression of *FLS* (*p* = 0.0031). Regarding temporal expression of flavonoid genes, *CH*S (*p* = 0.0001) and *ANS* (*p* = 0.028) were significantly higher at 24 hpi whiles *ANR* (*p* = 0.049) and *DF*R (*p* = 0.0110) were significantly higher at 48 hpi (Fig. 2a-e).

In this study, a total of 10 compounds belonging to different phenolic groups were identified and quantified. The content levels of the various classes and individual phenolic compounds in healthy and *B.* *cinerea* inoculated wild blueberry phenotypes are presented.

### Flavanols

Total flavanol which represents the sum of catechin and procyanidin B2 in this study was significantly (*p* = 0.0011) affected by *B. cinerea* infection (Table 1). Brown stem *Va* had a significantly higher flavanol content after 96 hpi compared to its control. Although there was a significant effect among the phenotypes, there was a wide variation in flavanol concentration between the healthy and inoculated plants among the various phenotypes. Given this, the flavanol concentrations in most of the phenotypes at the two time points were not significantly different from each other and their respective controls (Table 2).

**Table 1:** Total flavanols, hydroxycinnamic and flavonols subclasses of phenolic compounds (mg/g FW) in *B. cinerea* inoculated and healthy wild blueberry flower tissues

	***Va* brown stem**		***Va* green stem**		***Va* f. Nigrum**		***Vm* short stem**		***Vm* medium stem**		***Vm* tall stem**	
	**48 hpi**	**96 hpi**	**48 hpi**	**96 hpi**	**48 hpi**	**96 hpi**	**48 hpi**	**96 hpi**	**48 hpi**	**96 hpi**	**48 hpi**	**96 hpi**
**Total flavanols**												
**Control**	67.0 ± 10.2 abc	44.9 ± 8.1 efg	44.3 ± 3.7 efg	43.9 ± 6.3 efg	52.1 ± 16.2 b-e	54.6 ± 15.4 b-e	64.3 ± 12.3 a-d	67.9 ± 21.9 ab	59.8 ± 10.0 a-e	53.5 ± 7.1 b-e	57.5 ± 8.9 b-e	47.7 ± 7.7 b-f
***Botrytis cinerea***	60.8 ± 9.4 a-e	69.3 ± 9.8 ab	47.8 ± 4.3 d-g	31.3 ± 4.4 fg	46.1 ± 9.9 d-g	30.1 ± 15.0 g	76.7 ± 21.1 a	53.1 ± 4.8 b-e	45.2 ± 12.4 efg	49.0 ± 12.3 c-g	53.7 ± 13.4 b-e	46.2 ± 5.7 d-g
**Total hydroxycinnamic acid**												
**Control**	375.9 ± 113 abc	396.4 ± 120 abc	337.8 ± 13 a-d	361.6 ± 34.3 abc	408.7 ± 108 a	358.3 ± 19.1 a-d	270.1 ± 37.9 b-h	240.6 ± 65.7 d-h	266.3 ± 160 c-h	196.1 ± 122 e–h	195.6 ± 80 e–h	227.9 ± 60.8 d-h
***Botrytis cinerea***	324.5 ± 54.3 a-e	398.1 ± 62.7 ab	392.1 ± 26.7 abc	291.9 ± 95.7 a-g	348.2 ± 63.5 a-d	311.9 ± 91.6 a-f	328.8 ± 64.9 a-d	243.0 ± 54.5 d-h	189.3 ± 37.5 fgh	240.4 ± 32.3 d-h	179.1 ± 98.6 hg	157.1 ± 79.9 h
**Total flavonols**												
**Control**	113.1 ± 29.9 a-d	77.0 ± 30.3 b-f	112.2 ± 6.2 a-d	103.3 ± 7.7 a-e	71.6 ± 20.4 c-f	101.2 ± 10.7 a-f	101.9 ± 41.8 a-f	88.6 ± 52.3 b-f	93.6 ± 10.1 b-f	89.8 ± 20.2 b-f	66.3 ± 24.3 def	65.4 ± 22 def
***Botrytis cinerea***	117.8 ± 39.5 abc	147.2 ± 64.8 a	121.6 ± 3.2 ab	114.7 ± 10.6 a-d	67.9 ± 25.9 def	69.9 ± 22.2 def	110.4 ± 33.7 a-d	86.9 ± 23.1 b-f	56.1 ± 13.5 f	72.5 ± 10.9 c-f	60.6 ± 19.8 ef	67.3 ± 27.4 def

Mean value (*n* = 3) ± standard deviation. Mean separation was completed using least significant difference (LSD) test procedure. For each compound (across columns and rows), means with the same letters are not significantly different from each other at α = 0.05. Total flavanols: *P* = 0.0011, hydroxycinnamic acid: *P* = 0.0010, total flavonols *P* = 0.0156

Total flavanols is the sum of catechin and procyanidin B2

Total hydroxycinnamic acids is the sum of caffeic, chlorogenic neochlorogenic acids, m-coumaric acid and p-coumaric acid

Total flavonols is the sum of quercitin-3-galactoside, quercitrin and kaempferol-3-glucoside

**Table 2:** Concentration of individual flavanol compounds (mg/g FW) in *B. cinerea* inoculated and healthy wild blueberry flower tissues

	***Va* brown stem**		***Va* green stem**		***Va* f. Nigrum**		***Vm* short stem**		***Vm* medium stem**		***Vm* tall stem**	
	**48 hpi**	**96 hpi**	**48 hpi**	**96 hpi**	**48 hpi**	**96 hpi**	**48 hpi**	**96 hpi**	**48 hpi**	**96 hpi**	**48 hpi**	**96 hpi**
**Catechin**												
**Control**	33.2 ± 4.3ab	21.1 ± 5.0 c-f	18.5 ± 2.0 def	19.7 ± 3.0 def	21.8 ± 7.6 c-f	28.6 ± 10.7 a-d	30.7 ± 6.4 abc	32.1 ± 13.2 ab	27.6 ± 2.7 a-e	28.5 ± 5.7 a-d	23.0 ± 3.4 b-f	21.7 ± 3.1 b-f
***Botrytis cinerea***	30.8 ± 5.3 abc	35.2 ± 2.7 a	20.3 ± 2.2 def	15.9 ± 2.5 f	20.0 ± 7.1 def	14.5 ± 12.8 f	35.5 ± 8.5 a	24.1 ± 0.9 b-f	17.8 ± 4.4 ef	24.0 ± 3.2 b-f	21.5 ± 5.9 c-f	18.7 ± 3.5 def
**Procyanidin B2**												
**Control**	33.8 ± 6.3 a-d	23.7 ± 4.3 def	25.8 ± 1.7 b-f	24.2 ± 3.3 c-f	30.3 ± 8.9bcd	26.0 ± 5.1 b-e	33.6 ± 6.1 a-d	35.8 ± 8.9 ab	32.1 ± 8.8 a-d	25.0 ± 1.4 c-f	34.5 ± 5.7 abc	26.0 ± 5.1 bcd
***Botrytis cinerea***	30.0 ± 5.3 bcd	34.1 ± 7.1a-d	27.5 ± 2.2 bcd	15.3 ± 2.0 f	26.1 ± 3.8 b-e	15.6 ± 4.0 ef	41.2 ± 13.3 a	28.9 ± 4.7 bcd	27.4 ± 8.9 bcd	24.9 ± 9.2 c-f	32.2 ± 7.5 a-d	27.5 ± 4.9 bcd

Mean value (*n* = 3) ± standard deviation. Mean separation was completed using least significant difference (LSD) test procedure. For each compound (across columns and rows), mean with the same letters are not significantly different from each other at α = 0.05. Catechin: *P* = 0.0009, and Procyanidin B2: *P* = 0.0041

A significant difference in the concentrations of catechin (*p* = 0.0009) and procyanidin B2 (*p* = 0.0041) among the inoculated and healthy plants was observed. Similar to the total flavanol, there were higher concentrations of catechin in brown stem *Va* at 96 hpi, in the inoculated plants (Table 2). Like the total flavanol, most of the phenotypes either healthy or inoculated were not different from each other.

### Hydroxycinnamic acids

Hydroxycinnamic acid derivatives, which comprised the sum of caffeic, chlorogenic, neochlorogenic acids, m-coumaric acid, and p-coumaric acid were significantly affected by *B. cinerea* inoculation (*p* = 0.0010) (Table 1). Interestingly, the healthy *Va* f. nigrum had the highest concentration of hydroxycinnamic acids at 48 hpi although it was not different from most of the phenotypes either inoculated or uninoculated.

Chlorogenic acid characterized the majority (> 95%) of hydroxycinnamic acids measured. Changes in the concentration of chlorogenic acid (*p* = 0.0009), neochlorogenic acid (*p* = 0.0335) and m-coumaric acids (*p* < 0.0001) were detected among the treatments and phenotypes (Table 3). Although differences were observed, almost all the phenotypes were not different from each other. It is however worth noting that short *Vm* had a higher content of neochlorogenic acid in inoculated plants at 48 and 96 hpi (Table 3). The concentration of m-coumaric acid was higher in all inoculated *Va* phenotypes at different times of assessment except *Va f.* nigrum at 48 hpi. A higher concentration of m-coumaric acid was observed in inoculated short stem *Vm* and tall stem *Vm* at 48 and 96 hpi, respectively. No significant changes in the concentrations of caffeic acid and p-coumaric acid were observed.

**Table 3:** Concentration of individual hydroxycinnamic acid derivatives (mg/g FW) in *B. cinerea* inoculated and healthy wild blueberry flower tissues

	***Va* brown stem**		***Va* green stem**		***Va* f. Nigrum**		***Vm* short stem**		***Vm* medium stem**		***Vm* tall stem**	
	**48 hpi**	**96 hpi**	**48 hpi**	**96 hpi**	**48 hpi**	**96 hpi**	**48 hpi**	**96 hpi**	**48 hpi**	**96 hpi**	**48 hpi**	**96 hpi**
**m-Coumaric acid**												
**Control**	0.80 ± 0.3 efg	0.65 ± 0.2 fg	0.69 ± 0.3 fg	0.45 ± 0.1 g	0.95 ± 0.2 d-g	0.51 ± 0.1 g	0.70 ± 0.2 fg	0.82 ± 0.2 efg	0.94 ± 0.3 d-g	1.16 ± 0.5 b-f	1.53 ± 0.7 abc	0.63 ± 0.2 fg
***Botrytis cinerea***	1.38 ± 0.1 a-d	1.55 ± 0.5 abc	1.51 ± 0.4 abc	1.11 ± 0.2 c-f	1.48 ± 0.1 a-d	1.68 ± 0.1 ab	1.81 ± 0.5 a	1.34 ± 0.a-e	1.44 ± 0.3 a-d	1.32 ± 0.2a-e	1.60 ± 0.4 abc	1.80 ± 0.5 a
**Neochlorogenic acid**												
**Control**	3.87 ± 2.1 b-g	2.76 ± 0.2 g	3.10 ± 0.2 efg	2.86 ± 0.1 fg	3.48 ± 2.3 c-g	4.18 ± 2.1 b-g	5.74 ± 2.0 abc	5.45 ± 2.6 a-e	4.50 ± 2.2 b-g	5.67 ± 0.5 a-d	4.94 ± 0.6a-g	4.51 ± 0.8 b-g
***Botrytis cinerea***	3.21 ± 0.6 d-g	4.47 ± 2.4 b-g	1.95 ± 0.2 efg	2.36 ± 0.5 c-g	2.97 ± 1.1 efg	3.46 ± 1.8 c-g	7.38 ± 0.8 a	6.53 ± 0.1 ab	4.39 ± 1.5 b-g	5.28 ± 2.3 a-f	4.76 ± 0.6 b-g	5.02 ± 1.0 a-g
**Chlorogenic acid**												
**Control**	368.0 ± 114 abc	389.3 ± 121 ab	331.2 ± 13.2 a-d	355.6 ± 34.3 a-d	400.7 ± 109 a	349.6 ± 17.5a-d	259.9 ± 35.3 b-h	231.1 ± 62.7d-h	257.8 ± 162 c-h	186.7 ± 122 e–h	185.8 ± 79.4e-h	219.9.6 ± 89.8 d-h
***Botrytis cinerea***	316.9 ± 54.4a-e	387.4 ± 64abc	384.6 ± 26.8abc	283.8 ± 95.3a-g	340.3 ± 64.2a-d	302.7 ± 92.1 a-f	315.9 ± 63.2 a-e	233.0 ± 52.5d-h	180.7 ± 37.0fgh	229.9 ± 34.5d-h	169.4 ± 96.8 gh	146.4 ± 78.3 h
**Caffeic acid**												
**Control**	2.01 ± 0.4	2.71 ± 1.2	1.95 ± 0.1	2.05 ± 0.1	2.60 ± 0.8	2.66 ± 1.1	2.09 ± 0.3	1.87 ± 0.1	1.93 ± 0.2	1.77 ± 0.2	1.99 ± 0.3	1.72 ± 0.2
***Botrytis cinerea***	1.93 ± 0.3	2.65 ± 0.5	2.10 ± 0.3	2.34 ± 0.3	2.58 ± 0.7	2.74 ± 1.2	2.15 ± 0.2	2.36 ± 0.4	1.83 ± 0.1	2.39 ± 0.5	1.90 ± 0.4	2.09 ± 0.2
**p-Coumaric acid**												
**Control**	1.19 ± 0.6	0.92 ± 0.6	0.86 ± 0.0	0.69 ± 0.0	1.00 ± 0.5	1.31 ± 1.1	1.68 ± 0.7	1.35 ± 0.2	1.06 ± 0.0	0.83 ± 0.1	1.40 ± 1.2	0.91 ± 0.8
***Botrytis cinerea***	1.12 ± 0.6	1.96 ± 1.0	0.78 ± 0.1	1.35 ± 0.1	0.94 ± 0.5	1.35 ± 0.5	1.58 ± 0.5	1.91 ± 0.2	0.97 ± 0.4	1.53 ± 0.5	1.39 ± 1.5	1.78 ± 1.3

Mean value (*n* = 3) ± standard deviation. Mean separation was completed using least significant difference (LSD) test procedure. For each compound (across columns and rows), mean with the same letters are not significantly different from each other at α = 0.05.m-Coumaric acid: *P* < 0.0001, Neochlorogenic acid: *P* = 0.0335, and Chlorogenic acid: *P* = 0.0009

### Flavonols

Total flavonol, which is comprised of the sum of quercitin-3-galactoside, quercitrin (quercetin-3-rhamnoside) and kaempferol-3-glucoside, were also significantly affected by *B. cinerea* inoculation (*p* = 0.0156) (Table 1) with inoculated brown stem *Va* at 96 hpi having the highest concentration (Table 1).

Among the individual flavonols, no significant changes in the concentrations of quercitin-3-galactoside and quercetin-3-rhamnoside were observed. Kaempferol-3-glucoside was higher in inoculated brown stem *Va* at 96 hpi. Although changes in the kaempferol-3-glucoside concentration were significant, most of the phenotypes were not different from each other, where inoculated plants did not indicate significant differences when compared to their respective healthy plants (Table 4).

**Table 4:** Concentration of individual flavonol compounds (mg/g FW) in *B. cinerea* inoculated and healthy wild blueberry flower tissues

	***Va* brown stem**		***Va* green stem**		***Va* f. Nigrum**		***Vm* short stem**		***Vm* medium stem**		***Vm* tall stem**	
	**48 hpi**	**96 hpi**	**48 hpi**	**96 hpi**	**48 hpi**	**96 hpi**	**48 hpi**	**96 hpi**	**48 hpi**	**96 hpi**	**48 hpi**	**96 hpi**
**Quercitin-3-Galactoside**												
**Control**	63.5 ± 35.3	65.7 ± 29.1	106.5 ± 6.1	94.9 ± 10.8	63.5 ± 20.1	57.6 ± 32.2	79.8 ± 34.9	67.2 ± 42.1	74.2 ± 11.6	69.5 ± 9.9	49.5 ± 20.9	49.9 ± 21.2
***Botrytis cinerea***	67.7 ± 49.9	99.2 ± 91.3	115.8 ± 3.1	108.2 ± 10.9	60.3 ± 25.4	62.9 ± 24.5	90.3 ± 23.6	67.5 ± 15.6	40.3 ± 11.7	52.2 ± 6.9	44.7 ± 18.3	49.9 ± 24.3
**Quercitrin**												
**Control**	38.1 ± 39.3	7.01 ± 4.3	3.34 ± 0.1	5.89 ± 4.5	4.65 ± 1.1	32.0 ± 28.2	19.7 ± 8.3	19.7 ± 9.2	15.9 ± 4.3	14.4 ± 10.8	14.5 ± 9.6	13.4 ± 8.5
***Botrytis cinerea***	36.8 ± 34.2	36.2 ± 34.3	3.39 ± 0.1	3.81 ± 0.1	4.40 ± 1.4	4.71 ± 1.5	17.7 ± 9.7	16.2 ± 7.6	10.6 ± 1.1	11.6 ± 1.4	13.8 ± 10.0	14.1 ± 8.5
**Kaempferol-3-Glucoside**												
**Control**	11.5 ± 6.1 a	4.26 ± 1.7 bc	2.35 ± 0.2 c	2.50 ± 1.1 c	3.49 ± 1.3 c	11.5 ± 3.0 c	2.46 ± 1.0 c	1.73 ± 1.3 c	3.52 ± 1.4 c	5.87 ± 3.8 bc	2.23 ± 1.5c	2.08 ± 1.3 c
***Botrytis cinerea***	13.4 ± 6.4 a	11.7 ± 6.6 a	2.35 ± 0.1 c	2.62 ± 0.1 c	3.15 ± 1.6 c	2.33 ± 1.1 c	2.39 ± 0.7 c	3.26 ± 1.0 c	5.29 ± 2.9 bc	8.68 ± 4.1 ab	2.15 ± 1.4 c	3.28 ± 1.4 c

Mean value (*n* = 3) ± standard deviation. Mean separation was completed using least significant difference (LSD) test procedure. For each compound (across columns and rows), mean with the same letters are not significantly different from each other at α = 0.05. Kaempferol-3-Glucoside: *P* < 0.0001

## Discussion

In this study, we examined selected candidate genes that had previously been reported in literature to be expressed after pathogen infection. Generally, PR proteins have been reported to be induced in plants during pathogen attacks to improve host plants defense capacity [28–30]. Both *PR3* and *PR4* are genes that encode chitinases, which are known to play an important role in plant defense machinery by catalyzing the hydrolysis of chitin, a key structural component of fungal cell walls [31–33]. In plants, chitinases play a role in their development through their involvement in combating environmental stresses [34, 35]. Given the functions of chitinases, it is not surprising that many studies have reported that chitinase encoding genes (*PR3* and *PR4*) are up-regulated during host–pathogen interaction [13, 36, 37]. The early expression of *PR3* and *PR4* genes in the *Va* phenotypes, as well as the short and medium stem *Vm* phenotypes in this study agrees with previous studies [19, 38]. For instance, Koskimäki et al. [19] reported the accumulation of *PR4* genes in *V. myrtillus* 12 h after inoculation with *B. cinerea*. Although both *PR3* and *PR4* were weakly induced in this study, the expression of *PR4* was relatively high suggesting that *PR4* might play an important role in the defense of wild blueberry, especially *Va* f. nigrum against *B. cinerea*. The early and relatively high expression of these *PR* genes in *Va* f. nigrum among the phenotypes could partly explain the tolerance of *Va* f. nigrum to Botrytis blight compared to the other *Va* phenotypes [6].

Blueberry plants are a rich source of flavonoids and hydroxycinnamic acids such as flavonols, kaempferol, quercetin, catechins, and caffeic acid, chlorogenic acid respectively. These compounds perform several functions including the protection of plants against harmful radiation and plant defense against pathogens [39]. The biosynthesis of these compounds occurs in the phenylpropanoid pathway and changes in their accumulation are affected by the transcription profiles of genes such as *CHS*, *FLS*, *DFR*, *ANR*, and *ANS*. This study reveals that most of the flavonoid biosynthesis genes had similar expression patterns upon pathogen infection. Many studies have investigated the response of these flavonoid pathway genes in different plants [19 38]. Rose plant infected with *Podosphaera pannosa* and *Diplocarpon rosae* led to the upregulation of *CHS*, *FLS*, *DFR* and *ANS* [40]. Also, Cedar-apple plant infected with *Gymnosporangium yamadai* resulted in the upregulation of *CHS*, *FLS*, *DFR* and *ANS* [41]. Similar up-regulation of *CHS*, *DFR*, *ANS* and *ANR* was reported in *B. cinerea* infected bilberry [19]. Results from this study were in some cases consistent with these previous studies. For instance, compared to Koskimäki et al. [19] the up-regulation of *CHS, FLS*, *DFR, ANS* and *ANR* in this study was minimal, thus the up-regulation following a downregulation in some cases were below or similar to the basal expression levels. Similar to Lu et al. [41], there was an initial decrease in transcript levels of *CHS*, *FLS* and *ANS* in almost all the phenotypes at 12 hpi. The early decrease in the expression of flavonoid genes in this study could partly be attributed to the circadian rhythm in the plants. Ni et al., [42] indicated that circadian rhythms affected the flavonoid contents in Ginkgo leaves, where transcriptome results revealed a decrease in flavonoid gene expression in samples collected in the night. In this study, it is important to note that the 12 hpi samples were collected in the night (9 -10 pm), which could potentially explain the consistent decrease in the expression of the flavonoid genes at 12 hpi.

In addition to the flavonoid pathway genes, this study aimed to explore whether *B. cinerea* infection leads to changes in phenolics as part of the wild blueberry defense mechanism. Variation in the concentration of phenolic compounds in *B. cinerea* inoculated and healthy plants revealed differential behavior which is compound and phenotype dependent. The accumulation of phenolic compounds in plants, especially flavonoids as a component of defense mechanism against pathogens has been described by many studies [19, 43]. Mikulic‐Petkovsek et al. [44] found that *Didymella applanata* Sacc. and *Leptosphaeria coniothyrium* Sacc. infected raspberry increased specific phenolic compounds, such as flavanols. In Santin et al. [45], *Monilinia fructicola* Honey infected peach resulted in increased total phenolics and flavonols. Koskimäki et al. reported that *B. cinerea* infected bilberry contained higher levels of flavanols, flavonols and hydroxycinnamic acids [19]. Also, Keller et al. reported a high concentration of soluble phenolic compounds (derivatives of quercetin and hydroxycinnamic acid) in the calyptra of grape flowers after *B. cinerea* infection [46]. Furthermore, a phytotoxic sesquiterpene produced by *B. cinerea*, was found to induce the accumulation of reactive oxygen species and phenolic compounds in *Arabidopsis thaliana* [47]. Finally, Iwaniuk and Lozowicka found that stress caused by *B. cinerea* increased phenolic compounds in leafy vegetables [48].

Flavonoids are important compounds in blueberries [49, 50], and many studies have reported their accumulation and role as physiological regulators, chemical messengers, and inhibitors against biotic and abiotic stress [41, 51]. Inoculation of wild blueberry flowers with *B. cinerea* resulted in the accumulation of some flavanols, flavonols and hydroxycinnamic acid in this study. The individual phenolic compounds, particularly *m*-coumaric acid, and kaempferol-3-glucoside were the compounds that were increased with *B. cinerea* inoculation. The results of this study agree with previous findings of phenolic compound accumulation in infected plants, particularly flavonols and flavanols [42, 52–54]. Interestingly, some of the hydroxycinnamic acids had decreased concentration in infected plants. Nonetheless, these finding corroborates the report of some previous studies [44, 55]. This observation in hydroxycinnamic acids may be due to their naturally high abundance in blueberry or their role as a substrate in the biosynthesis of some complex phenolics, such as lignin and suberin [56]. Hydroxycinnamic acids, particularly chlorogenic acid were the most abundant phenolic observed in this study which may suggest that they form part of pre-formed biochemical defense in wild blueberry. Given their abundance, a further increase in their concentration during pathogen attacks might not be essential.

Molecular and plant defense response events can be triggered by a variety of abiotic or biotic factors. Given that this study was conducted under field conditions and on a perennial plant, the wild blueberry plants were in constant interaction with the environment, which may account for the relatively low levels of gene expressions and seeming fluctuation pattern for some of the genes and phenolic compounds (Supplementary file, Figure S1). Studies have demonstrated that in the field, plants are partly induced through their interaction with both biotic and abiotic factors. Pasquer et al. [57] found that the expression of defense genes was already at a high level in wheat plants before the application of defense elicitors (benzo (1,2,3) thiadiazole-7-carbothioic acid S-methylester, BTH) under field conditions. Also, Herman et al. [58] found that different cultivars exhibited near-baseline expression levels of defense genes when plants were initially induced with acibenzolar*-S*-methyl (ASM). Furthermore, given the induction of flavonoid genes in bilberry by the endophyte, *Paraphaeosphaeria* sp. [19], one will not rule out their potential contribution to the variation in flavonoid gene expression observed. Additionally, environmental factors such as light and temperature have been reported as important elements that affect flavonoid pathway genes [59, 60]. Azuma et al. [61] reported that low temperature and light have a synergistic effect on the expression of genes that are involved in flavonoid biosynthesis. Given the complexity of the environment and the perennial nature of the plants, the major determinant of this variation cannot be easily identified. Nonetheless, it is worth noting that despite the basal expression of these defense and flavonoid genes, some of the genes were significantly upregulated over the different time points, suggesting the potential involvement of these genes in wild blueberry plant defense against *B. cinerea*.

The variation in the phenolic response in this study could be due to natural variation in the field and environmental conditions. Environmental factors such as light, radiation and temperature have been reported to affect secondary metabolism in fruits including *Vaccinium* spp. [62]. The variation in phenolic compounds is not surprising because many studies have also reported significant phenolic variation within and among different cultivars [44, 50]. Although the difference between infected and healthy plants was observed for some compounds, phenolic changes among the various phenotypes mostly did not show any statistical significance as observed with the flavonoid genes. The accumulation of flavonoids is governed by a complex network of genes in the phenylpropanoid pathway and regulatory genes [12], hence, under such complex study conditions, similarity in the variation between the flavonoid genes and the flavonoid compounds is noteworthy.

Results from this study reveal a difference between the expression levels and response time among the phenotypes, indicating a phenotype-specific response mechanism to the pathogen. The more susceptible *Va* phenotypes responded to pathogen infection earlier (mostly at 12 hpi) than *Vm*, which mostly showed upregulation at 24 hpi. Interestingly, this finding contradicts previous research, which found that resistant cultivars exhibit early responses with mostly high levels of defense-related genes upon pathogen infection [63, 64]. The reason for this is unknown, however, this could partly be related to *Vm’s* morphological and physical features. *Vm* is covered with pubescence/hair-like structures [65], which have the potential to interfere with direct plant surface contact by conidia. This could potentially delay pathogen perception and defense response activation in *Vm*. Although there was a difference in the gene expression pattern, the transcript levels among the various phenotypes did not indicate any statistical significance. One reason might be the low expression levels observed. In addition, the wide variation observed on wild blueberry fields, even within the same phenotypes could contribute to the non-significance observed among the phenotypes. Although *Vm* and *Va* phenotypes had similar expression values, it is worth noting the difference in ploidy between the two groups. Polyploid species tend to have higher expression of genes during genome analysis [66, 67]. Hence, coupled with its unique morphological features and late flower bud development, theoretically doubling the expression levels in the *Vm* phenotypes could show strong up-regulation of the various genes to possibly explain why *Vm* is less susceptible to pathogens.

## Conclusion

Understanding the molecular mechanism employed by wild blueberry against *B. cinerea* infection is important for sustained wild blueberry production and the development of disease control tools. In this study, the infection of wild blueberry by *B. cinerea* was characterized by phenotype-specific increased expression of *PR* genes which suggests their potential involvement in wild blueberry defense machinery. Additionally, a most common response of downregulation of flavonoid genes was observed followed by a weak upregulation. Also, our results indicate that the induction and accumulation of phenolic compounds in *B. cinerea* infected flowers might be temporal and phenotype dependent. This study may provide insight into the wild blueberry defense mechanism and serve as a starting point for achieving a better understanding of the wild blueberry-*B. cinerea* pathosystem and the path to incorporate induced resistance as defense strategies in wild blueberry production.

## Supplementary Information

**Additional file 1: Table S1.** List of primer pairs used for gene expression studies. **Table S2.** List of phenolic compounds, their retention times and wavelength of determination. **Figure S1.** Environmental conditions (Leaf wetness, temperature, and rainfall) observed in Benvie Hill, NS in June, 2019. X: High risk *Botrytis* infection period, +: Moderate risk *Botrytis* infection period.

## Data Availability

All data generated or analysed during this study are included in this published article [and its supplementary information files].
