# Supplementary material for: Modulation of defense genes and phenolic compounds in wild blueberry in response to Botrytis cinerea under field conditions
Source: BMC Plant Biol. 2023 Feb 28;23:117. doi: 10.1186/s12870-023-04090-5 (PMC9972761; doi:10.1186/s12870-023-04090-5)
Supplement: Supplementary file 1 — Additional file 1: Table S1. List of primer pairs used for gene expression studies. Table S2. List of phenolic compounds, their retention times and wavelength of determination. Figure S1. Environmental conditions (Leaf wetness, temperature, and rainfall) observed in Benvie Hill, NS in June, 2019. X: High risk Botrytis infection period, +: Moderate risk Botrytis infection period. [file 12870_2023_4090_MOESM1_ESM.docx]

**Supplementary file**

**Table S1.** **List of primer pairs used for gene expression studies.**

| **Gene name** | **Accession number** | **Primer** | **Sequence details (5’-3’)** | **Amplicon size** |
| --- | --- | --- | --- | --- |
| *PR3* | MK292726 | Forward | AGACTTGGTAGCAACTGAC | 82 |
|  |  | Reverse | GGAAGGTTTCGGGGATTG |  |
| *PR4* | MK292724 | Forward | TAACTACAACCCGGAGCAGG | 164 |
|  |  | Reverse | GCAAGCACTTCCCACAAGAA |  |
| *CHS* | MK333527 | Forward | TCCCAGATCAAGAAGAGGTACA | 119 |
|  |  | Reverse | ATTTCCACAACCACCATATCCT |  |
| *FLS* | MK333531 | Forward | CTCCTTCTTACAGGGAAGCTAATG | 79 |
|  |  | Reverse | GACAGCCACTTGAACAACTTG |  |
| *ANS* | MK333528 | Forward | GAATCACCTGAGAGCCCTAAC | 75 |
|  |  | Reverse | AGCCTGTCTTCTTCCAATCC |  |
| *ANR* | MH321471 | Forward  Reverse | CAAAGACCCTAGCGGAGAAAG | 98 |
|  |  |  | GGAGAAACACCAGCCATAAGA |  |
| *DFR* | MK333524 | Forward  Reverse | CTGCTGGAACCGTCAATGT | 139 |
|  |  |  | GCTGCTTTCTCTGCTAGTGTT |  |
| *GAPDH* | AY123769 | Forward | CAAACTGTCTTGCCCCACTT | 207 |
|  |  | Reverse | CAGGCAACACCTTACCAACA |  |

**Table S2. List of phenolic compounds, their retention times and wavelength of determination**

| **Compound** | **Retention time (min)** | **Wavelength (λ)** |
| --- | --- | --- |
| Catechin | 25.30 | 280 |
| Procyanidin B2 | 25.84 | 280 |
| *m-*coumaric acid | 28.93 | 280 |
| *p-*coumaric acid | 28.22 | 302 |
| Chlorogenic acid | 26.17 | 302 |
| Caffeic acid | 26.72 | 302 |
| Neochlorogenic acid | 22.69 | 302 |
| Quercetin-3- galactoside | 29.20 | 355 |
| Quercetrin (Quercetin 3-rhamnoside) | 30.00 | 355 |
| Kaempferol -3- glucoside | 30.07 | 355 |

**Figure S1. Environmental conditions (Leaf wetness, temperature, and rainfall) observed in Benvie Hill, NS in June, 2019. X: High risk *Botrytis* infection period**, **+: Moderate risk *Botrytis* infection period**
